# Qualitative study on the use of emergency services by people with serious mental disorder in Spain

**DOI:** 10.1186/s12875-023-02078-6

**Published:** 2023-06-20

**Authors:** Alejandro Pérez-Milena, Juan Andrés Ramos-Ruiz, Natalia Zafra-Ramirez, Carmen Noguera-Cuenca, Antonina Rodríguez-Bayón, Beatriz Ruiz-Díaz

**Affiliations:** 1 “El Valle” Primary Care Center, Andalusian Public Health System, 4 Human Rights Street, 23009 Jaén, Spain; 2 Multiprofessional Teaching Unit of Family and Community Care Jaén North – Northeast, Andalusian Public Health System, Linares, Spain; 3 Multiprofessional Teaching Unit of Family and Community Care Jaén - South Jaén, Andalusian Public Health System, Jaén, Spain; 4 Department of Psychology, University of Almería, Almería, Spain; 5 Multiprofessional Teaching Unit of Family and Community Care North – Northeast Jaén, Andalusian Public Health System, Jaén, Spain

**Keywords:** Emergency medical services, Mental disorders, Family support, Rural population, Qualitative research

## Abstract

**Background:**

The population with severe mental disorders (SMD) is a frequent user of emergency services. Situations of psychiatric decompensation can have devastating consequence and can cause problems in getting urgent medical care. The objective was to study the experiences and needs of these patients and their caregivers regarding the demand for emergency care in Spain.

**Methods:**

Qualitative methodology involving patients with SMD and their informal caregivers. Purposive sampling by key informants in urban and rural areas. Paired interviews were carried out until data saturation. A discourse analysis was conducted, obtaining a codification in categories by means of triangulation.

**Results:**

Forty-two participants in twenty-one paired interviews (19 ± 7.2 min as mean duration). Three categories were identified. 1º Reasons for urgent care: poor self-care and lack of social support, as well as difficulties in accessibility and continuity of care in other healthcare settings. 2º Urgent care provision: trust in the healthcare professional and the information patients receive from the healthcare system is crucial, telephone assistance can be a very useful resource. 3º Satisfaction with the urgent care received: they request priority care without delays and in areas separated from the other patients, as well as the genuine interest of the professional who attends them.

**Conclusions:**

The request for urgent care in patients with SMD depends on different psychosocial determinants and not only on the severity of the symptoms. There is a demand for care that is differentiated from the other patients in the emergency department. The increase in social networks and alternative systems of care would avoid overuse of the emergency departments.

**Supplementary Information:**

The online version contains supplementary material available at 10.1186/s12875-023-02078-6.

## Background

The National Healthcare System in Spain has more than 50 million requests for emergency care each year. These are met in three organizational areas: primary care, mobile emergency services and hospital emergency departments (EDs). In Spain, anyone can choose freely and without economic cost what type of medical care they want to receive according to their subjective perception of symptoms severity. As a result, the average emergency department visits have been increasing over the last three decades [1, 2]. More than 50% of the Spanish population went to an emergency medical service in 2019 [3], mostly due to banal pathology [4]. Only during the first wave of the COVID-19 pandemic this number decreased generally [5].

The population with severe mental disorder (SMD) is a social group with greater demand for urgent medical attention than the general population, despite being assigned one or more mental health network resources [6–8]. Psychiatric emergencies have been defined as acute disturbances of thought, behaviour, mood or social relationships that require immediate intervention as defined by the patient, the family or the social unit to save the patient and/or other people from imminent danger [9]. Situations of psychiatric decompensation can have devastating consequences for the ill people and their families, which is why it is a priority to stabilize the patient with SMD and prevent suicidal ideation [10]. However, most of the situations treated in the emergency services have not presented this seriousness and could have been resolved through routine medical care or better health education [6–8, 11].

Crowded emergency medical services may compromise patient treatment satisfaction, contributing to increased health care costs promoting the appearance of high emergency department users [11]. We face a complex situation in which the demand for emergency care among this population is conditioned not only by the physical and mental health status, but also for a wide range of socioeconomic and demographic factors that cannot be addressed exclusively by the organization of emergency services [12]. People at risk of social exclusion, with high rates of poverty and unemployment, and those who live in rural areas, with social isolation, decide to go to the emergency services more frequently [13]. Similarly, the care provided by family members to people with MDS in Spanish society is very important and decisive when choosing to go to an emergency department [7].

Knowledge of the current socioeconomic context, the family support and the biopsychosocial circumstances that accompany and cause the frequentation of the emergency department by the population with SMD would make it possible to rethink the healthcare offer and better adapt the resources [12]. The objective of this study was to gain a holistic understanding of the needs of SMD patients and their main caregivers in situations requiring emergency medical care.

## Methods

### Study design

**Fig. 1:**
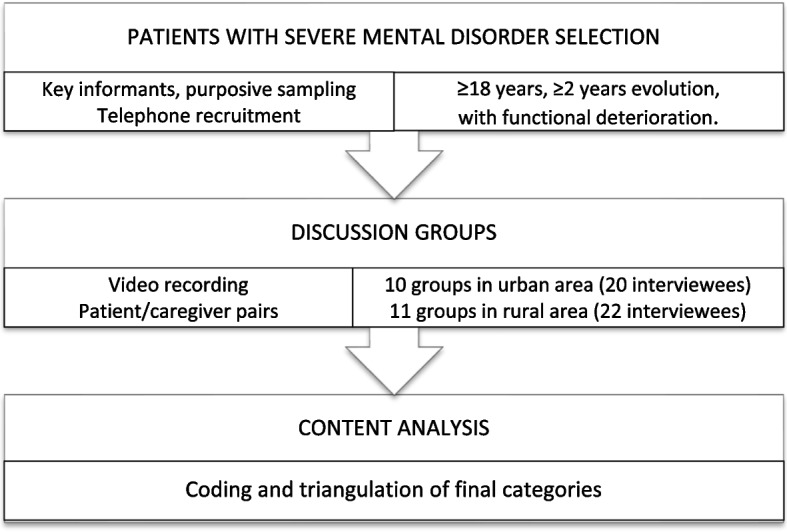
Flowchart of the qualitative study

An exploratory qualitative study with a holistic approach was performed to find and list the causes of the phenomenon following the Standards for Reporting Qualitative Research (SRQR) recommendations (Fig. 1).

### Sampling and recruitment

The fieldwork was carried out from January to September 2021 in two Basic Healthcare Areas. One area was urban with 12,000 inhabitants with 40% at risk of social exclusion, and a second rural area considered to be geographicaly isolated with difficult access to healthcare, with 6,250 inhabitants (7 villages with < 1,000 people).

The study population consisted of patients with a diagnosis of SMD (Table 1) and the family member providing informal care. Diagnostic criteria include all psychotic disorders, excluding the organic ones, including not only the presence of positive and negative symptoms, but also a severely disturbed pattern of relationships, inappropriate behavior according to the context or severe inappropriate affectivity, involving a distorted perception of reality. The patients included had a disease course of more than 2 years and a progressive and pronounced deterioration in functioning over the last 6 months, even if the symptoms had subsided. Patients were included if they were at least 18 years old and had attended at least two non-urgent medical consultations over the last year. Patients living in closed institutions or foster homes, the ones with no permanent address and those cared for by formal caregivers, were excluded.

**Table 1:** Diagnostic categories of the International Classification of Diseases (ICD-10) for severe mental health disorder people included in the study

- Schizophrenic disorders (F20.x)
- Schizotypal disorder (F21)
- Persistent delusional disorders (F22)
- Shared psychotic disorders (F24)
- Schizoaffective disorders (F25)
- Other psychotic disorders not due to a substance or known psychosocial condition (F28 and F29)
- Bipolar disorder (F31.x)
- Major depressive episode with psychotic features (F32.3)
- Recurrent major depressive disorders (F33)
- Obsessive–compulsive disorder (F42)

Sampling was purposive, cumulative and sequential, with a family doctor from each Basic Healthcare Area acting as key informant, supporting themselves by the opinion of other health professionals such as nurses and social workers and results from a previous study [13]. The groups were treated as homogeneous when both patient and caregiver were present at the interview. Heterogeneity criteria were gender, diagnosis, degree of disability of the patient (assessed using the WHODAS 2.0 questionnaire for Disability Assessment of the WHO), perception of family dysfuntion (Apgar-Family test) and the caregiver’s sense of overload (Zarit burden scale). It was initially estimated that 8 interviews would be carried out in rural areas and 8 in urban areas (16 patients and 16 caregivers), but this was extended to 11 in towns and 10 in the city to achieve information saturation (21 patients and 21 caregivers) (Fig. 1).

### Data collection

The information was collected through conversational techniques using focus group methodology. The interviews were moderated by a non-health professional, videotaped and transcribed verbatim. The total number of interviews was subject to the saturation of discourse. Data saturation was reached when there is enough information to replicate the study, when the ability to obtain additional new information has been attained, and when further coding is no longer feasible.

### Data analysis and interpretation

The texts obtained were evaluated by means of a three-phase qualitative content analysis. Phase 1 involved the coding, i.e. viewing the recordings and the literal reading of the transcripts, generation of the first pre-analytical hypotheses and confirmation of data saturation. Phase 2 involved the category triangulation with the identification and grouping of the text units and their referent discourses into categories agreed apon by the researchers. In phase 3 the results were obtained and verified. Matrices were constructed with the support of NVivo software and the final categories were defined.

#### Ethics approval and consent to participate

Ethical approval was previously obtained by means of a review provided by the Research Ethics Committee of the Andalusian Healthcare Service (TMG01 protocol, 2019/03/28) (Jaén provincial committee, Spain), with reference to ethical principles of the Helsinki Declaration. Participants were reminded that their participation was completely voluntary and were requested to sign informed consent forms. To protect the participants’ anonymity, no personal information was disclosed. Videotape recordings were deleted according the ICC/ESOMAR International Code.

## Results

**Fig. 2:**
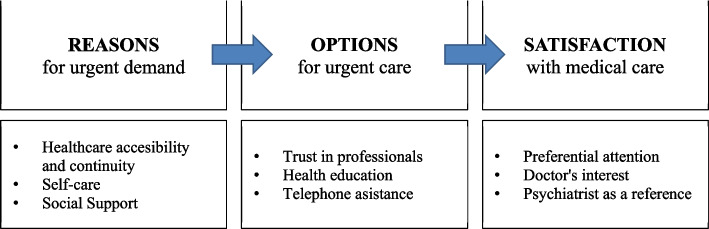
Relationships between the main categories and subcategories

Twenty-one patient/caregiver paired interviews have been carried out with a total of 42 participants and a mean duration of 19 min (± SD 7.2) (range 5–33 min). The patients had diagnoses of schizophrenia (38%), bipolar disorder (31%) and major depression (31%), with a mean age of 58.8 years (± SD 13.1), 63% were women, 32% were disabled and 25% had poor family function. The caregivers were first-degree family members (50% spouses, 31% sons and/or daughters, 13% siblings and 6% parents), 53% were men and the mean age was 52.9 years (± DS 16.9), 60% showed caregiving burden and 32% perceived family dysfunction. The characteristics according to an urban or a rural area are shown in Table 2. The data collected is presented in three categories and nine subcategories (Fig. 2 and Table 3) corresponding to the topics of the interview script, the hypotheses generated and the explanatory framework.

**Table 2:** Main characteristics of the people interviewed according to the study area

	**Rural area**		**Urban area**	
	**Patient**	**Caregiver**	**Patient**	**Caregiver**
Duration of the interview	21,8 ± 2,5 min		16,8 ± 9,7 min	
Gender (female)	57,1%	66,7%	66,7%	33,3%
Average age (years)	62,7 ± 9,6	52,5 ± 15,4	55,8 ± 15,1	53,2 ± 18,9
Family dysfunction (Family APGAR Questionnaire.)	42,9%	42,9%	11,1%	22,2%
Patient disability (WHODAS 2.0 Questionnaire)	28,6%	–	33,3%	–
Caregiver overload (Zarit burden scale)	–	83,3%	–	44,4%

**Table 3:** Verbatims for each category and subcategory

**Category 1: Reasons for urgent assistance**
A. Self-care. *“The psychiatrist told me that if I ever feel bad, I should go there right away (to Mental Health), although I try to hold on.”* (Woman with bipolar disorder and disability, urban area)
B. Self-care. *“You don’t know how to act when he’ s unwell. No matter how hard I fight, I have no answers and no one comes to help me.”* (Female caregiver with intense overload, rural area)
C. Social support. *“My experience in the village is not very gratifying. There is very little access to psychiatric consultations and emergency services; you have to travel for everything. At the moment, I find myself alone.”* (Female caregiver with intense overload and family dysfunction, rural area)
D. Social support. *“I’m very frightened by this COVID thing, I’m stuck at home all day. I go out as little as possible, I find myself more lonely and I am very stressed.”* (Man with depressive disorder and disability, urban area)
E. Accessibility and continuity of care. *“There’s a long wait for a checkup and when you need it the most, they’re not there. So, you have to go to the emergency department.”* (Female caregiver with intense overload, urban area)
F. Accessibility and continuity of care*. “I’ve put on weight because of the pandemic and because of the naps. And he doesn’t sleep at all, I need to rest. It’s awful, and without an appointment I can’t consult anyone.”* (Female caregiver with intense overload, urban area)
**Category 2: Urgent care options**
G. Trust. *“When you go to the emergency room, you are attended by young people, who you don’t know, but they are very nice, very friendly. We have been lucky to have met some very, very nice doctors”* (Female caregiver with intense overload, urban area)
H. Information on the health care system. *“I go to the Public Health Insurance as well as to the private sector. They treat him there as if he were family, you know…”* (Female caregiver with poor family function, rural area)
I. Telephone assistance*. “It would be very good if we had fast access to care, a telephone number to speak directly to the psychiatrist.”* (Woman with bipolar disorder, urban area)
J. Telephone assistance*. “I need face-to-face consultations because I don’t have enough time to explain myself over the phone and to say all the things that are happening to me”* (Man with schizophrenic disorder and disability, urban area)
K. Telephone assistance *“He (the private psychiatrist) assisted me several times over the phone and stayed on the phone as long as I needed. He resolved all the doubts I had and assisted me very well.”* (Male caregiver, urban area)
**Category 3: Satisfaction with the urgent care received**
L. Priority in assistance*. “The waits are agonizing, I find it very difficult, I have to go outside. You spend a lot of hours waiting and they don’t call you.”* (Woman with bipolar disorder and disability, urban area)
M. Professional’s interest*. “Then when you do go in (after having waited) and you only get five minutes (in the consulting room)… The doctor needs to be attentive, take a close look at your medication, chat a bit and let you tell him your doubts".* (Woman with borderline personality disorder and major depression, urban area)
N. Symptom-focused solutions. “*They give you a tranquilizer, and at least you stop having that paranoia. Then you need to be seen by your psychiatrist in the consulting room*.” (Woman with bipolar disorder and disability, urban area)

### Reasons for urgent care

#### Self-care

Some SMD patients have been informed by their psychiatrist about when to request an urgent assessment (Table 3A). This information is more useful when patient and caregiver have sufficient capacity to detect alarm symptoms and when there is adequate compliance with the pharmacological treatment. The criteria used by caregivers to request an urgent assessment is influenced by the presence of new psychopathological symptoms as well as caregiving overload, the perception of poor functionality of the family dynamics and greater disability of the patient (Table 3B). The lack of proactive help from the healthcare system increases the caregiver's uncertainty and promotes the use of an emergency department, especially in rural areas.

#### Social support

Other family and social conditioning factors create the need for urgent consultation, such as social isolation or social interaction difficulties. This is more pronounced in rural populations due to hospital remoteness and the social stigma of this disease (Table 3C). Similarly, the feeling of helplessness and isolation of patients and caregivers has increased during the pandemic due to home confinement and the restriction on social interactions (Table 3D).

#### Accessibility and continuity of care

Delays in routine primary care lead to emergency department consultations, even without the appearance of new symptoms (Table 3E). This situation has worsened during the first periods of the COVID-19 pandemic, with a feeling of greater vulnerability in this population due to the worsening of the symptoms in patients and the appearance of anxiety, depression and insomnia in caregivers (Table 3F). Similarly, the lack of communication between family physicians and psychiatrists results in distrust in the former to go to Primary Care when faced with a problem considered urgent.

### Urgent care options

#### Trust

The options used by patients and families when facing the need for an urgent consultation are to go to their Mental Health Team, to be evaluated in the emergency services or to go to private healthcare. The choice is based on the trust they put in the professionals of each area. Patients and caregivers trust their psychiatrists the most since they are well-known professionals who usually provide friendly, respectful and confidential treatment. Secondly, there are good expectations regarding care in hospital emergency departments, with the aim of getting a fast, although provisional solution to the problem until the next Mental Health check-up (Table 3G).

#### Information from the healthcare system

In urban areas patients know how to reach their mental health team in emergency situations, which is not the case in rural areas. This lack of information forces them to visit the emergency services more frequently. Caregivers in villages turn to private medicine, which offers continuity of care by the same psychiatrist and several possibilities to contact for urgent problems (Table 3H).

#### Telephone attention

There are considerable differences in the accessibility of and satisfaction with telephone consultations as a form of urgent care. Especially patients with less disability and more family support request a telephone line to consult a professional, even if not a psychiatrist, who can advise them on how to act in emergency situations (Table 3I). For patients with greater disabilities and exhausted caregivers, however, urgent telephone assessment impairs the quality of human contact and hinders verbal communication.

They experience less accurate professional assessment (Table 3J) since they are forced to select the reasons for consultation and shorten the time of the clinical meeting. Regarding these experiences, patients and caregivers who use private medicine value telephone care very highly as a first urgent contact, as it allows the psychiatrist to make it possible to prioritize the face-to-face consultation and to adjust the medication immediately (Table 3K).

### Satisfaction with medical care

#### Priority of care

All the discourses concur regarding requesting immediate attention upon arrival at the hospital emergency department. They experience excessive waiting times before being attended and want to be prioritized over other less important problems (Table 3L). Staying in the waiting room with the other patients is a recurrent negative experience, although they do understand the difficulties of the healthcare staff due to the saturation of the emergency services and the lack of human resources in the healthcare system.

#### Professional’s interest

The closeness and interest of the professionals working in the emergency department increase overall satisfaction after being attended, even with professionals other than psychiatrists or those with less experience. Both patients and caregivers show the need for professional assessment but also for someone lending a sympathetic ear and a holistic knowledge of the biopsychosocial aspects (Table 3M).

#### Symptom-focused solutions

The solutions obtained in the emergency department are temporary and helpful for reducing symptoms, but patients and caregivers wait for the psychiatrist in charge to confirm or not the changes that have been made (Table 3N).

## Discussion

The urgent demand for care arises after a self-diagnosis made by the patient with SMD and/or his or her caregiver when noticing loss of health. This urgent need for care depends not only on symptoms related to mental health, but also on the quality of self-care, accessibility and continuity of healthcare and social support [14, 15]. Equally important is the knowledge of the range of health resources available to these patients who have socially normalized the use of hospital emergency departments due to the lack of health education and a culture where people are used to receiving immediate care in a hospital [4, 15, 16]. Going to an emergency department for lack of other resources would compensate for the inequalities and social inequities experienced by this population, but it does not take into account ethical or economic aspects, underestimates the importance of the decision of healthcare professionals [12] and does not ensure adequate continuity of care [17].

The feeling of isolation and lack of resources is much more pronounced in rural than in urban areas [13]. Loneliness within the family and society results from the loss of social ties due to the stigma of the mental illness, causing family and social networks to become less close and more fragile, which overburden the caregiver. Institutional isolation and the lack of information on health resources is greater than in urban areas and create a reality of contexts to be intervened by social and healthcare services [13]. Avoiding unnecessary visits to the emergency department involves improving accessibility and outpatient monitoring, with a more accessible healthcare network to deal with unexpected situations [18]. In this sense, telemedicine is a possible and accepted approach for this population that would help to address the urgent needs of patients, families and communities, especially in rural areas [19, 20]. Spain has already had successful experiences with the introduction of preferential telephone care services for people with SMD, which have improved communication and access to mental health resources. Caregivers do not ask for direct and exclusive access to the psychiatrist, but rather to a professional who can guide them in difficult times and facilitate continuity of care in Mental Health. This type of telematic care has shown greater efficiency and accessibility, reduction of waiting times and unnecessary psychiatric hospitalizations along with high levels of patient satisfaction [20, 21].

Despite adequate training of the physicians to attend to urgent situations [22], improvements in staffing levels and structural and care network reforms of the hospital EDs over the past few decades [6, 12, 23], there are still problems of overcrowding due to the increase in urgent demand. In the case of the population with SMD, the hospital response cannot completely resolve all the needs detected [16, 23]. Nevertheless, some aspects, such as waiting times for medical care or prioritizing care over other trivial problems, would improve the perception of the quality of emergency care [24].

Qualitative study limitations may difficult to to generalise the results to other settings with different cultural and socio-economic characteristics. The results obtained from a convenience sample would only apply to populations with similar characteristics to the ones that have been studied. Opinions of health professionals, institutionalized patients or those who live alone were not collected due to the objective of the study and the characteristics of the recruitment. The results obtained may be affected by the possible presence of a selection bias, by not including all diagnostic categories of severe mental disorders, and a social desirability bias.

Nevertheless, it is important to highlight the experiences of people in rural areas and the ones at risk of social exclusion because of their greater vulnerability and higher rates of emergency service use [12, 13, 24]. On the other hand, the social health situation generated by the COVID-19 pandemic provides a different panorama in a population that is more vulnerable but hesitant to go to the emergency department out of fear of contagion [5, 25]. The pandemic situation has modified the circumstances of emergency care, generating greater saturation and delays which can lead to dissatisfaction and violation of the patient’s privacy [12].

The management measures adopted from the emergency services themselves have been insufficient to reduce demand for urgent medical care [7–9]. For this reason, it is necessary to carry out studies on the usefulness of the collaborative coordination in care transitions between emergency services and primary care [23] and the innovation for medical access through use of technology as telephone consultations [26, 27]. Future research should study the impact of improving complex social elements [28], such as emotional support for caregivers or increasing the close social network, in order to improve self-care of this population [29]. It is equally important to know the efficiency of improving the early detection of mental pathology, an additional support systems and the access to alternative healthcare resources for this population group as more efficient options for resolving situations perceived as urgent [9, 12, 26, 30].

## Conclusions

Reducing unnecessary emergency department visits should be a goal of all primary care and mental health services. Unnecessary urgent medical care demand burden the health care system as they are costly and consume resources that other individuals with more acute needs may need. Experiences and needs of patients with SMD and their caregivers, both in terms of prompt care and in terms of proximity, confidentiality and privacy, are the key to knowing the areas for improvement. In addition to changes in healthcare management, it is a priority to promote community participation to assess and address social determinants of health and health disparities. These options include the implementation of community resources, the personalized support for caregivers and the improvement of social cohesion networks. The experiences and needs revealed in this study offer a community diagnosis that would allow the adaptation of the supply to the real needs of emergency care and promote solutions without relying exclusively on emergency services. A holistic approach of different multidisciplinary solutions to manage emergencies in patients with SMD would promote rational use of healthcare resources and better healthcare outcomes.

## Supplementary Information

**Additional file 1.**

## Data Availability

Study data are transcripts of interviews that are not publically available due to concerns that participants’ privacy may be compromised. Anonymized and de-identified data may be requested from the corresponding autor.

## Abbreviations

COVID-19: Coronavirus Disease of 2019
EDs: Emergency Departments
SMD: Severe Mental Disease
SRQR: Standards for Reporting Qualitative Research
WHO: World Health Organization
WHODAS: WHO Disability Assessment Schedule

## References

[1] Oterino de la Fuente D, Baños Pino JF, Fernández Blanco V, Rodríguez Álvarez A y Peiró S (2007). Hospital and primary care emergency services in Asturias[Spain]: variations among health areas and trends between 1994–2001. Gac Sanit.

[2] Torné Vilagrasa E, Guarga Rojas A, Torras Boatella MG, Pozuelo García A, Pasarin Rua M, Borrell Thió C (2003). Analysis of demand in the emergency services of Barcelona. Aten Primaria.

[3] Activity and Quality of Health Services. Annual Report of the National Health System 2017. Ministry of Health, Government of Spain. 2017. https://www.mscbs.gob.es/estadEstudios/estadisticas/sisInfSanSNS/tablasEstadisticas/InfAnualSNS2017/5_CAP_17.pdf. Accessed 9 Dec 2022.

[4] Wai AK, Chor CM, Lee AT, Sittambunka Y, Graham CA, Rainer TH (2009). Analysis of trends in emergency department attendances, hospital admissions and medical staffing in a Hong Kong university hospital: 5-year study. Int J Emerg Med.

[5] Montero-Pérez FJ, Jiménez Murillo LM (2021). Impact of the first COVID-19 pandemic wave on the care and quality indicators of a hospital emergency department. Emergencias.

[6] Tudela P, Mòdol JM (2015). On hospital emergency department crowding. Emergencias.

[7] Guerra Mora P, Bouza Fustes R, Concha V, Ibáñez López C, López Noche MM (2021). Hiperfrequency in mental health hospital emergencies: case-control study. Rev Esp Salud Publica.

[8] Muñoz López M, Panadero Herrero S, Rodríguez González A, Pérez SE (2009). Evaluation of care for people with serious and persistent Mental illness: the Madrid experience. Clínica y Salud.

[9] Basterra-Gortari V (2017). Demand for emergency for care Spanish adults with a current or past history of mental health problems. Emergencias.

[10] Wheat S, Dschida D, Talen MR (2016). Psychiatric emergencies. Prim Care.

[11] Dolan MA, Fein JA, Committee on Pediatric Emergency Medicine (2011). Pediatric and adolescent mental health emergencies in the emergency medical services system. Pediatrics.

[12] Moreno ME (2008). What if we adapted hospital emergency services to social demand and not to health needs?. Emergencias.

[13] Ramos-Ruiz JA, Pérez-Milena A, Enguix-Martínez N, Álvarez-Nieto C, Martínez-Fernández ML (2013). Community diagnosis using qualitative techniques of expectations and experiences in a health area in need of social transformation. Aten Primaria.

[14] Ortega Maján MT, Rabanaque Hernández MJ, Júdez Legaristi D, Cano Del Pozo MI, Abad Díez JM, Moliner Lahoz J (2008). Profile of users and reasons for demand of the 061 extra-hospital Emergency Service. Emergencias.

[15] Expósito-Ruiz M, Sánchez-López J, Ruiz-Bailén M, Rodríguez-del Águila MM (2017). Factors related to the use of pediatric emergency services: results from the Spanish National Health Survey. Emergencias.

[16] Armoon B, Cao Z, Grenier G, Meng X, Fleury MJ (2022). Profiles of high emergency department users with mental disorders. Am J Emerg Med.

[17] Urbanoski K, Cheng J, Rehm J, Kurdyak P (2018). Frequent use of emergency departments for mental and substance use disorders. Emerg Med J.

[18] Saeed SA, Diamond J, Bloch RM (2011). Use of telepsychiatry to improve care for people with mental illness in rural North Carolina. N C Med J.

[19] Reinhardt I, Gouzoulis-Mayfrank E, Zielasek J (2019). Use of telepsychiatry in emergency and crisis intervention: current evidence. Curr Psychiatry Rep.

[20] Salmoiraghi A, Hussain S (2015). A systematic review of the use of telepsychiatry in acute settings. J Psychiatr Pract.

[21] Butterfield A (2018). Telepsychiatric evaluation and consultation in emergency care settings. Child Adolesc Psychiatr Clin N Am.

[22] Nable JV, Lawner BJ, Brady WJ (2016). 2016: emergency medical services annotated literature in review. Am J Emerg Med.

[23] Pines JM, Hilton JA, Weber EJ, Alkemade AJ, Al Shabanah H, Anderson PD (2011). International perspectives on emergency department crowding. Acad Emerg Med.

[24] Boyle A, Beniuk K, Higginson I, Atkinson P (2012). Emergency department crowding: time for interventions and policy evaluations. Emerg Med Int.

[25] Marroquín B, Vine V, Morgan R (2020). Mental health during the COVID-19 pandemic: effects of stay-at-home policies, social distancing behavior, and social resources. Psychiatry Res.

[26] Gråwe RW, Ruud T, Bjørngaard JH (2005). Alternative emergency interventions in adult mental health care. Tidsskr Nor Laegeforen.

[27] Fleury MJ, Grenier G, Bamvita JM, Caron J (2011). Mental health service utilization among patients with severe mental disorders. Community Ment Health J.

[28] Fleury MJ, Ngui AN, Bamvita JM, Grenier G, Caron J (2014). Predictors of healthcare service utilization for mental health reasons. Int J Environ Res Public Health.

[29] Toot S, Devine M, Orrell M (2011). The effectiveness of crisis resolution/home treatment teams for older people with mental health problems: a systematic review and scoping exercise. Int J Geriatr Psychiatry.

[30] Baraff LJ, Janowicz N, Asarnow JR (2006). Survey of California emergency departments about practices for management of suicidal patients and resources available for their care. Ann Emerg Med.

