# Supplementary material for: Qualitative study on the use of emergency services by people with serious mental disorder in Spain
Source: BMC Prim Care. 2023 Jun 20;24:125. doi: 10.1186/s12875-023-02078-6 (PMC10280892; doi:10.1186/s12875-023-02078-6)
Supplement: Supplementary file 1 — Additional file 1. [file 12875_2023_2078_MOESM1_ESM.docx]

**INTERVIEW GUIDE for FOCUS GROUP**

**QUALITATIVE STUDY ON THE USE OF EMERGENCY SERVICES BY PEOPLE WITH SERIOUS MENTAL DISORDER IN SPAIN**

Pérez-Milena A, Ramos-Ruiz JA, Zafra-Ramirez N, Noguera-Cuenca C, Rodríguez-Bayón A, Ruiz-Díaz B.

**Greeting and Welcome**

Introduce moderator.

*“My name is… My role as moderator will be to guide the discussion. Thank you for agreeing to participate in this interview, your opinion is very important to us*.”

**Communicative Agreement**

Presentation of the research group.

*“I represent a group of doctors and psychologists who work in different health centers in the province of Jaén. We are carrying out an investigation and we need your personal opinion on various health issues. We are very grateful for your participation today in this interview.”*

Purpose of the interview and explanation of the study objective.

*“We would like to know the experiences of people who have a serious mental health problem, as well as their family caregiver, in situations that require urgent medical attention. Above all, we want to know your needs when urgent health problems arise and the experiences when it is necessary to go to an emergency medical department. The results will be used for improving urgent medical care for people with severe mental disease.”*

Interview setting, duration and confidentiality.

*“The interview will last from 20 to 30 minutes. You can speak in order or comment among yourselves. This interview will be recorded on video and then it will be transcribed in writing. We are concerned about your privacy: no personal data will be disclosed and the video recordings will be destroyed. Please, ask any question about the study and leave the interview at any time.”*

*Interview rules are:*

- *No right or wrong answers, only differing points of view*
- *We're tape-recording, one person speaking at a time*
- *You don't need to agree with your relative, but you must listen respectfully to his/her points of view .*

**In-depth discussion**

Starter question.

*“Our topic is ‘experiences and needs in urgent medical care’. I imagine that you have ever had to go to a medical emergency service due to a worsening of your illness. How was the experience?”*

Opening question. Allow interviewees to talk freely with each other.

- Situations that require urgent medical attention.
- Healthcare accesibility and continuity in Primary Care.
- Quality of emergency medical care.
- Trust in healthcare professionals.
- Knowledge of duties and rights of the patient.
- Necessary improvements in the organization of urgent medical care.

Emerging topics: note to moderator.

- Collect new topics that arise for subsequent interviews.

**Closing of the interview**

Making a brief summary of the contributions.

Final contributions.

"Before finishing I would like to know if you want to add anything else...".

Thank the participation.

“We appreciate your participation, in delivered documents (informed consent) you have our contact address in case you need to contact us. Thank you so much.".
